# How to Measure Sedentary Behavior at Work?

**DOI:** 10.3389/fpubh.2019.00167

**Published:** 2019-07-05

**Authors:** Gil Boudet, Pierre Chausse, David Thivel, Sylvie Rousset, Martial Mermillod, Julien S. Baker, Lenise M. Parreira, Yolande Esquirol, Martine Duclos, Frédéric Dutheil

**Affiliations:** ^1^Faculté de Médecine, Institut de Médecine du Travail, Université Clermont-Auvergne, Clermont-Ferrand, France; ^2^Cellule d'Accompagnement Technologique–Department of Technological Accompaniment, CNRS, LaPSCo, Université Clermont Auvergne, Clermont-Ferrand, France; ^3^Laboratory of the Metabolic Adaptations to Exercise Under Physiological and Pathological Conditions (AME2P EA 3533), Université Clermont Auvergne, Clermont-Ferrand, France; ^4^Institut Universitaire de France, Paris, France; ^5^Unité de Nutrition Humaine, INRA, Université Clermont Auvergne, Clermont-Ferrand, France; ^6^LPNC, CNRS, Université Grenoble Alpes, Université Savoie Mont Blanc, Grenoble, France; ^7^School of Science and Sport, Institute of Clinical Exercise and Health Sciences, University of the West of Scotland, Hamilton, United Kingdom; ^8^Occupational and Preventive Medicine, INSERM UMR-1027, Université Paul Sabatier Toulouse 3, CHU Toulouse, Toulouse, France; ^9^Sport Medicine and Functional Explorations, CRNH, INRA UMR-1019, University Hospital of Clermont-Ferrand, Université Clermont Auvergne, CHU Clermont-Ferrand, Clermont-Ferrand, France; ^10^LaPSCo, Physiological and Psychosocial Stress, Preventive and Occupational Medicine, CNRS, University Hospital of Clermont-Ferrand, Université Clermont Auvergne, CHU Clermont-Ferrand, WittyFit, Clermont-Ferrand, France; ^11^Faculty of Health, School of Exercise Science, Australian Catholic University, Melbourne, VIC, Australia

**Keywords:** occupational health, sedentary lifestyle, workplace, sedentary behavior measurement, work, questionnaires, wearable devices, recommendations

## Abstract

**Background:** Prolonged sedentary behavior (SB) is associated with increased risk for chronic conditions. A growing number of the workforce is employed in office setting with high occupational exposure to SB. There is a new focus in assessing, understanding and reducing SB in the workplace. There are many subjective (questionnaires) and objective methods (monitoring with wearable devices) available to determine SB. Therefore, we aimed to provide a global understanding on methods currently used for SB assessment at work.

**Methods:** We carried out a systematic review on methods to measure SB at work. Pubmed, Cochrane, Embase, and Web of Science were searched for peer-reviewed English-language articles published between 1st January 2000 and 17th March 2019.

**Results:** We included 154 articles: 89 were cross-sectional and 65 were longitudinal studies, for a total of 474,091 participants. SB was assessed by self-reported questionnaires in 91 studies, by wearables devices in also 91 studies, and simultaneously by a questionnaire and wearables devices in 30 studies. Among the 91 studies using wearable devices, 73 studies used only one device, 15 studies used several devices, and three studies used complex physiological systems. Studies exploring SB on a large sample used significantly more only questionnaires and/or one wearable device.

**Conclusions:** Available questionnaires are the most accessible method for studies on large population with a limited budget. For smaller groups, SB at work can be objectively measured with wearable devices (accelerometers, heart-rate monitors, pressure meters, goniometers, electromyography meters, gas-meters) and the results can be associated and compared with a subjective measure (questionnaire). The number of devices worn can increase the accuracy but make the analysis more complex and time consuming.

## Introduction

Sedentary behavior (SB), has been defined as sitting or lying with low energy expenditure ≤1.5 METs (1) and is an independent risk factor for numerous adverse health outcomes. In industrialized modern societies, more and more time is spent for SB activities during normal lifestyle behavior, such as working on computers, traveling by car, and watching television during leisure time (2, 3). Further to this, more workers are now employed in low activity jobs such as administrative work. Office workers can have SB for more than of their working day (4). Chronic disease and all-cause mortality have been linked with self-reported time spent sitting (5–13). A dose response relationship has been demonstrated between all-cause mortality and daily total sitting, with a 2% increase in all-cause mortality per hour seated per day (14). Even after adjustment on the quantity of moderate or vigorous physical activity (15, 16), the risk of death persists, demonstrating that time spent sitting is a risk factor independent of the level of physical activity. SB can be measured by declarative methods (auto-administrate questionnaires) and objective methods (observation, video, or technical instruments). Descriptive parameters of physical activity and sedentary activity used most often are duration, frequency, intensity, domain or context (leisure, work, domestic, transport), and the type of activity. Indicators combining these parameters can be calculated globally or for each one of the domains individually. The most common are the volume (time × frequency) and the energy expenditure (duration × frequency × intensity), the latter being calculated to account for overall physical activity. Time spent in front of a screen (television, video, video games, computer…) is currently the most used sedentary indicator and in the majority studies, is the time spent watching television measured by survey techniques. Considering the public health impact of SB at work, there is now a growing research interest about sedentariness at work. However, SB is measured through a wide range of methods, but no scientific articles provide a global overview on all methods used to quantify sedentary behavior.

## Objective

The aim of this paper was to provide a global understanding on methods currently available for SB assessment at work.

## Search Strategy

**Figure 1:**
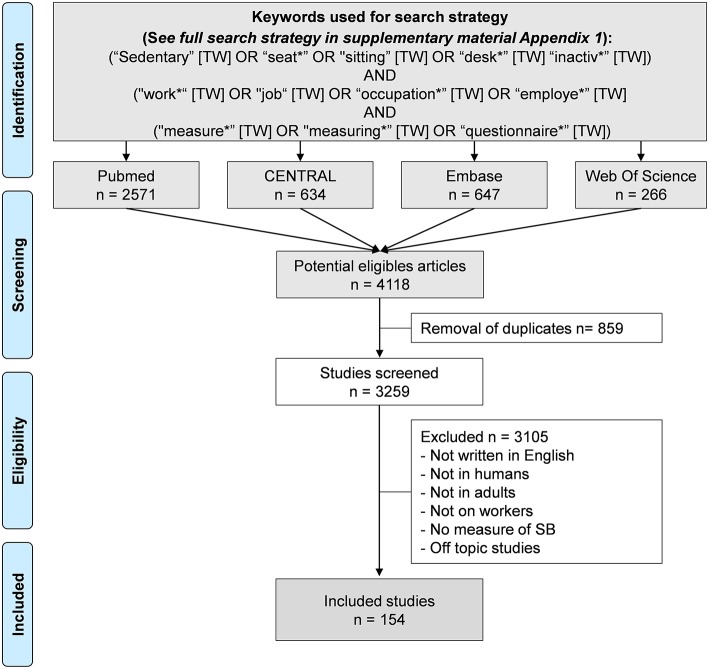
Search strategy.

Published studies with measures of SB at work were retrieved through a systematic search of the Pubmed, Cochrane, Embase, and Web of Science databases. We selected articles published between 1st January 2000 and 27th March 2019 because SB gained momentum in recent years, with more diversity on assessing SB at work, and because only recent articles distinguished between SB and physical inactivity and their specific health effects (6, 17–19). The search strategy and keywords used are detailed in Supplementary Material Appendix 1. We restricted our search to articles in humans and written in English. We did not restrict our search to specific countries or regions, nor on a minimal sample size. Included articles had to describe tools used to measure SB at work. The search strategy is displayed in Figure 1. Three authors (GB, PC, FD) conducted all literature searches and agreed on the final decision for articles inclusion. A fourth author (MD) reviewed articles when no consensus was met. Then, eligible articles were reviewed by all authors.

## Data Extraction and Synthesis

We extracted the following information: type of study (longitudinal, cross-sectional), category of material (questionnaire, one common sensor, multiple sensors, complex physiological system), number of subjects and the main measure of sedentariness. Identified devices which assessed sedentary behavior at work where tabulated to highlight the performance and the usability of methods and devices to access sedentary behavior at work (see Table S1 for the complete lists of included articles with those details).

## Characteristics of Included Articles

**Figure 2:**
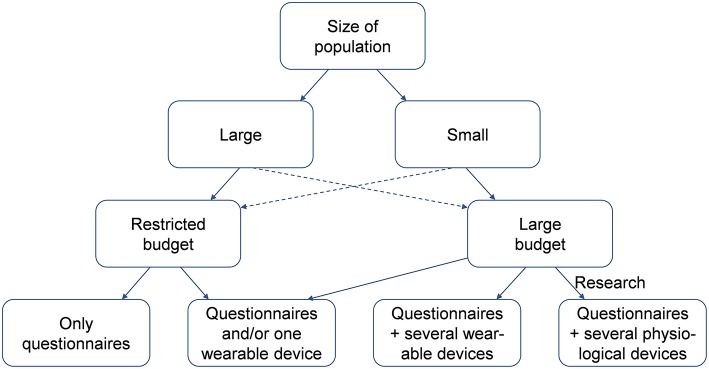
Decision strategy for the best option to measure sedentary behavior at work.

**Figure 3:**
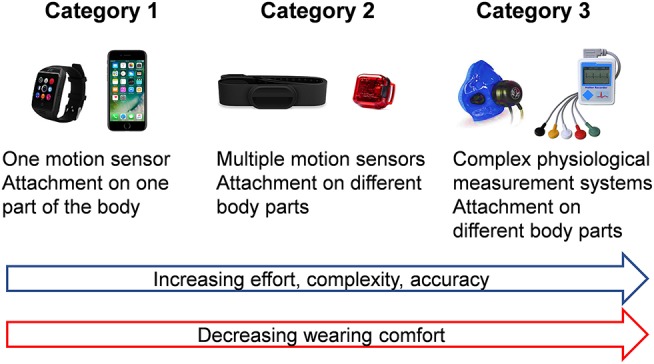
Categorization of wearable devices to measure sedentary behavior depending on accuracy and complexity.

An initial search retrieved a possible 4,118 articles. Removing duplicates and applying the selection criteria decreased the number of articles reporting measures of SB at work to 154 articles (Figure 1). Among the 154 included articles, 89 were cross-sectional studies, and 65 were longitudinal studies, for a total of 474 091 participants. SB was assessed by self-reported questionnaires in 91 studies, and by wearables devices in also 91 studies. Among those studies, 30 studies used simultaneously a questionnaire and wearables devices. Among the 91 studies using wearable devices, 73 studies used only one device, 15 studies used several devices, and three studies used complex physiological systems. Studies exploring SB on a large population used significantly more only questionnaires and/or one wearable device. Complete list of included articles, with details on the type of the study, number of participants, type of measures of SB, and main outcomes are presented in Table S1. Methods of measuring SB retrieved in included articles are detailed below. For practitioners and researchers who want to evaluate SB at the workplace, we propose a strategy for the best options to evaluate SB in the workplace, depending on several factors, including comfort, number of subjects, duration of measures, accuracy, and budget (Figures 2, 3 and Table 1).

**Table 1:** Instrument, raw unit, cost, and environment of tools to measure sedentary behavior at work.

**Instrument**	**Measure/raw unit**	**Cost**	**Environment**
Questionnaire	Response quote qualitative	Negligible	Possible at work but take time
Video observation	Video qualitative	50 to 500€ for a camera	May need authorization especially at work
Smartphone	All sensors (XYZ g, m/s, position, direction, brightness illuminance lux …)	300 to 1000€ + costs of applications	Easy to wear and common
Accelerometer	g or count (on X,Y,Z axis 3D, position, direction, brightness illuminance lux)	50 to 400€	Easy to wear even at work
Heart rate monitor	Beat/minute	50 to 300€	Easy to wear even at work
Holter-ECG	RR interval from ECG	300 to 2000€	Easy to wear even at work
Gas analyser	O2 CO2 consumption/production (liter, m^3^…)	20 to 30000€	For a short period on few individuals Less comfortable

## Methods of Measuring Sedentary Behavior

### Declarative Methods-Self-Reported Questionnaires

These questionnaires are the most common method of measuring SB, relying on recall ability of participants (20). The commonly used self-report questionnaires for SB at work assessment are: The Global Physical Activity Questionnaire (GPAQ), International Physical Activity Questionnaire (IPAQ) (21, 22), Workforce Sitting Questionnaire (WSQ, Adapted from the Marshall Questionnaire), Occupational Sitting and Physical Activity Questionnaire (OSPAQ) (23) and European Physical activity Questionnaire (EPAQ) (24). Questionnaires differed on global characteristics of SB or PA (such as duration, intensity or frequency), precision of data (habitual or recent, leisure, or non-leisure activities), reporting data (such as time, calories, or scores), time of recall (such as last week or over the 12 last months), and method for conducting the survey (such as paper, computer, face-to-face) (25). Questionnaires have the advantages of their low cost and low effort, both for responders and researchers, rendering them accessible for studies in large populations. However, self-reported SB at the workplace has been demonstrated to be imprecise, biased in measurement of light or moderate physical activity, and in the assessment of energy expenditure. Severe others limitations are the dependency on written language and external factors such as age, seasonal variation, complexity of the questionnaire, and social desirability) (26–30). Characteristics and performances of questionnaires for SB assessment at work are presented in Table 2.

**Table 2:** Characteristics of self-report questionnaires to measure sedentary behavior at work.

**Measure**	**Period(s) of interest**	**Categories of activity included**	**Input**	**Output**	**Special notes**
GPAQ	Typical week	16 items; PA at work, Moderate to vigorous, Transportation, Leisure-time	MET-min per week	Time spend in moderate or vigorous PA, Job-related PA, Total physical activity, Time spend sitting	For adults of both sex. For face-to-face interviews conducted by trained interviewers. Many domains explored. Quantifies exposure. Cross cultural application. 20 min.
IPAQ-S (short)	Past week	7 items; moderate or vigorous PA, walking, sitting, including time spend at work	Duration (min per week)	Duration in each PA domain and sitting, Job-related	For adults of both sex. Self-administered. Many settings and in different languages. Cross cultural application. Shorter than IPAQ-S. 10 min.
IPAQ-L (Long)	Past week	24 items; moderate or vigorous PA, walking, sitting, including Job-related PA, house work, transportation PA, and weekend	Duration (min per week)	Duration in each PA domain and sitting, Job-related, house work, leisure	For adults of both sex. Self-administered. Many settings and in different languages. Cross cultural application. 30 min.
WSQ (Workforce Sitting Questionnaire)	Past week	Duration of work. Total and domain-specific sitting time based on work and non-workdays, transportation. Time spend watching TV, computer, others leisure	Duration (min per week)	Duration of work. Time spend sitting at work and in non-workdays. Time spend in transportation, in screen watching and other leisure	For adults of both sex. Self-administered. For measuring sitting time at work on a work-day and for assessing total sitting time based on work and non-workdays. Cross cultural application.
OSPAQ	Past five working days	7 items; Work time spent sitting, standing, walking, and doing heavy labor, as well as the total length of time worked in the past five working days	Duration (min per week)	Time spend sitting, standing and walking, and doing heavy labor and total length of working	For adults of both sex. Self-administered. Only Job-related PA, excluding transportation, and leisure time. 10 min.
EPAQ	Typical week	21 items; Sitting and standing, moderate PA in leisure and working time, heavy labor at work	Duration (min per week)	Time spend standing, sitting, doing moderate PA at work and in non-workdays, in house work, and leisure and heavy labor at work	For adults of both sex. Self-administered. Do not distinguish moderate and vigorous PA, but focus on moderate PA. Assessed walking and bicycle separately.

*MET, Metabolic equivalent of task (1 MET represents 3.5 ml/kg/min oxygen consumption); PA, Physical Activity; Questionnaires, GPAQ Global activity Questionnaire, IPAQ International Physical Activity Questionnaire, IPAQ-S (Short version), IPAQ-L (Long version), WSQ (Workforce Sitting Questionnaire), EPAQ (European Physical Activity Questionnaire)*.

## Objective Methods

### Visual Observation (Direct or Videotaped)

SB at work can also be assessed by visual observation, either recorded or on-site. Visual observation is still a classical method used by ergonomics, occupational physicians, or researchers (30). This method of assessment is often use for assessing body postures at work in delimited space (e.g., work space). Contextual information (such as location, clothing, or time) and details on SB (such as type or personal activities) could be assessed with this method. However, direct observations are costly and time consuming (31), and are therefore mostly adapted for small populations and on short periods. Visual observations are also dependent on observers who may rate differently the same behavior (32). Observed workers may also modify their behavior (observational bias) because of the logistic burden associated with data collection. Videotaped monitoring at work also needs the authorization of the employers and workers and ethical consideration.

## Cardiorespiratory Assessment

### Indirect Calorimetry (IC)

With IC, total energy expenditure is calculated from Weir's formula that takes into account oxygen consumption and carbon dioxide production (33). This accurate and non-invasive method can be used in routine but not in large epidemiological studies nor for measures in an ecologic environment (outside of a laboratory or a specific workplace setting). Moreover, materials needed are costly. For data collection, the workers needs to wear a facemask linked with a central unit. For ambulatory measurements, the central unit could be worn in a backpack. Thus by discriminating energy expenditure, SB is defined as seated, reclining, or lying activities requiring low levels of energy expenditure (i.e., ≤1.5 METs), light-intensity physical activity (LPA) as standing is between 1.6 and 2.9 METs and Moderate- to vigorous-intensity physical activity (MVPA) require energy expenditure ≥3.0 METs). IC can evaluate sedentary time. These analyzers are now portable like the Cosmed K5 (34) or Metamax Cortex (35). Their use over a long period can be difficult to support depending on the activity of the worker but are still feasible. Because of the relatively slight differences in energy expenditure between sitting and standing posture (36, 37), assessment of energy expenditure does not provide reliable information about the body posture. So, measurement of body posture is also required for assessment of SB at work. Conversely, most of body positions at work can be assessed by wearable devices. The use of multiple devices may also inform on anatomical location of movements.

### Holter-Electrocardiography (Holter-ECG)

A linear relationship between cardiorespiratory response and energy expenditure, and thus with activity intensity has been clearly demonstrated (38). Heart rate (HR) can therefore be used to estimate energy expenditure. Coupling HR monitoring and accelerometers leads to a better accuracy in the assessment of SB and physical activity (30, 39). Historically, electrical HR sensors detect the electric impulses that are linked with the myocardial contraction. The signal allows detection of all heartbeats, and therefore of the HR. In clinical setting, the gold-standard for electrocardiographic assessment is a 12-lead ECG. In an ecologic environment (outside of hospital), a portable 3 or 5-lead Holter-ECG is commonly used for scientific research. It allows abnormal heart rhythms and cardiac symptoms detection and is considered as a medical device. Commercially wearables Holter-ECG are often based on simply a 1- or 2-lead ECG. Despite its accuracy and validity, measures with 1- or 2-lead Holter-ECG are more susceptible to artifacts because of external factors, and therefore are not consider as a medical device. Major causes are motion, physical and muscle activity, or detachment of electrodes (40, 41). To allow better diagnostic accuracy, the worker can place time markers for specific activities or events at the workplace. Data can be stored directly into a specific memory into the device or in a digital storage media (e.g., SD cards). Data are downloaded and analyzed with specific softwares by a cardiologist, a physician, or a researcher.

### Heart-Rate Monitors

There are two different types of technology used by HR monitors: the electrical signal (chest belt) and optical sensor (wristwatch or armband) (42). Chest belts detect electrical signals sent through the heart each time it contracts (ECG-based detection of RR interval). Sometimes, chest belts can transmit HR data on a wristwatch providing a feedback (pulse monitoring) to the user. The Optical HR measurement is based on photoplethysmography (PPG). The Optical HR devices use integrated LED and light sensors to detect HR through rhythmic changes in blood flow occurring at each systole (blood volume pulse) (43). These sensors are cheap, discrete, and comfortable. They are mostly placed on wrists and arms, and sometimes ear lobes or fingertips. Main limitations are artifacts because of motion and a decreased sensitivity with some skin texture (44). ECG-based chest belts still offer the most reliable, consistent, and accurate way to monitor HR thanks to higher sampling rates and the position of the electrodes closer to the heart (45). However, many people prefer the comfort and convenience of optical sensors built into watches, such as Applewatch. HR monitors are able to capture energy expenditure during working activities and to categorize levels of physical activity. Moreover, they can estimate the energy expenditure even with no vertical trunk displacement that is not taking into account by most accelerometers and pedometers (46). HR monitors are less accurate to estimate energy expenditure particularly at very high and low intensities (47), because the relationship between HR and energy expenditure is not linear for high intensity of physical activity or at rest and low-intensity (with confounding factors such as body position, stress, or caffeine affecting the HR—energy expenditure relationship) (47). Others factors also affect this linear relationship or reduce its accuracy, such as age, sex, body composition and muscle mass, or fitness level (48).

### Accelerometers

Accelerometers are currently used to measure and quantify the physical activity intensity category related to SB and have become the method of choice for measuring SB. Accelerometers are easy to use, accurate, and able to capture large amounts of data, particularly in large studies. These devices detect movement in real time and measure acceleration (counts) in three orthogonal planes (anteroposterior, mediolateral, and vertical) (49). The postulate is that the acceleration detected is proportional to the force produced by the muscles engaged in motion, and therefore related to energy expenditure. Time of SB is assessed by two different ways to detect body posture (standing, sitting, or lying): (1) posture by tri-axial sensors using gravitational components, or (2) spinal curvature by three uni-axial gyroscopes orthogonally aligned. Some accelerometers fail to differentiate walking intensity or body position (such as standing or sitting) (50). New accelerometers have a better validity than older models, compared to energy expenditure measured by doubly labeled water (DLW). However, accelerometers cannot provide contextual information (such as type of activity and setting) and induce a reactivity bias (51). Accuracy to determine SB depends on the threshold chosen for each count (count cut-point) (52). Most of the time, the acceleration counts characterize sedentary (absence of movement) and active behavior. The most commonly used cut-points for adult populations are <100 counts/min for SB, 100–1,951 counts/min for light-intensity physical activity (LPA), and ≥1,952 counts/min for moderate- to vigorous-intensity physical activity (MVPA) for the ActiGraph accelerometer (53, 54). However, these cut-points were developed in specific populations and during strict, laboratory-based protocols. Other studies validating the ActiGraph have found vastly different cut-points for SB (range 50–250 counts/min) and MVPA (191–2,691 counts/min) in adults, depending on the population and type of validation setting (55, 56). The cut-point method has several limitations; it cannot differentiate standing from sitting/lying, but standing is considered LPA because it elicits different physiologic responses and has different long-term health consequences than sitting/lying (57, 58). Thus, the interpretation of what is considered to be active behavior is consequently different and makes the comparison between the studies difficult. Obese people spend more time in SB than normal weight individuals (59, 60). Thus, cut-points have to be more accurate to show difference among and between normal-weight and obese populations. Accelerometers worn on the right thigh achieve high accuracy for classification of three distinct physical activity intensity categories (SB, LPA, and MVPA) as well as breaks in SB in a semi-structured setting. Wrist accelerometers also have high accuracy for assessment of SB but have some misclassifications of LPA and MVPA, with interestingly better accuracy when they are worn on the left wrist compared to the right wrist (or hip). These findings support the use of accelerometers worn at the thigh to assess the time spent in SB and different categories of physical activity intensity. Alternately, for researchers using wrist-worn accelerometers to assess physical activity, wear on the non-dominant wrist is likely to allow for higher measurement accuracy than wear on the dominant wrist (61). Due to limitations of the cut-point approach to measure categories of physical activity intensity, researchers have utilized modelization technics to improve accuracy of physical activity measurement from accelerometers worn on various body locations (62, 63). An accelerometer does not give the position information of the subject. It will be completed by a gyroscope (measuring orientation and angular velocity) (Samsung Gear S3) and a magnetometer (detecting Erath's magnetic three perpendicular axes X, Y, Z) (Actigraph GT9X) (64). The ActivPal is an alternative tri-axial accelerometer thigh-worn. The thigh position allows the determination of step counts, stepping speed, and start-end of each period spent sitting, lying, standing, or stepping, as well as breaks in SB and postural transitions. The ActivPAL is a monobloc system that is discrete, easily used by individuals, without calibration, and reliable for the measurement of SB (65, 66). Therefore, ActivPAL is increasingly used in ecological environment outside laboratories.

### Global Positioning System (GPS)

Global Positioning System (GPS) can complete this variety of sensors by giving the geographical position (latitude and longitude) and time of each geographical position, but mainly outside building. Newer GPS can also deliver information such as speed (retrieved from time between different geographical positions), elevation, and indoor/outdoor activities. However, most workers spend a high proportion of their time indoors, and unfortunately GPS are only able to receive indoor signal from small buildings with a wooden roof or high buildings with large windows. GPS are unable to determine room-level of indoor location (67). However, even if GPS is mostly for outdoor activities, newest GPS can also track SB indoors. Moreover, some devices also include useful tools such as a brightness sensor to access sleep quality. These wearable lightweight GPS devices are easily forgotten by users. The researcher should take care to check the sampling frequency, resolution, and the maximum amplitude of the device. In order to make long observations, it is also necessary to check the device battery and storage space. Recent smart-phones or smartwatches are equipped with all the mentioned sensors.

### Smartwatches and Smartphones

Smartwatches are wrist-worn computerized devices with extensive communication capabilities. They are linked to one mobile operating system. In perpetual development, manufacturers continue to implement new features, such as GPS, fitness/health tracking, or waterproof frames (16). The gestures of the hands, such as smoking, are now accessible thanks to the addition of reliable and sensitive inertial sensors (17). In a recent meta-analysis (68) the most popular smartwatches (connected devices) on the market were compared: from Apple, Fitbit, Garmin, Lumo, Misfit, Samsung Gear, and TomTom. Generally, smartwatches tend to underestimate energy expenditure compared to laboratory reference measurements (Oxycon Mobile, CosMed K4b2, or MetaMax 3B). Moreover, while smartwatches get better to estimate energy expenditure with an increased intensity, validity becomes poorer with low intensity, and sedentary measures. Because everyone has a smartphone, they are an alternative to smartwatches or other wearable devices. Now, all smartphones combine many sensors, such as GPS or Global Navigation Satellite System (GLONASS), accelerometer, e-compass, gyroscope, proximity sensor, or ambient light sensor. Conveniently, smartphones can be linked with an HR belt, a smartwatch, or even a gas analyzer. However, all wrist and forearm devices have a tendency for underestimating HR, especially for exercises at high intensity and with amplitude of arm movement (such as exercising on a treadmill or an elliptical machine)—and conversely, measures of HR are more accurate at rest or for exercise without movement of arms (such as on a cycle ergometer). While HR is underestimated for high intensity of physical activity, step count on the opposite is underestimated for slower walking speeds and in free-living conditions. Smartphones are also particularly attractive for context awareness and phone-based personal information (69). The recognition of some activities are dependent on position-attachment of the phone on the body (70). For example, to recognize a specific activity, the smartphone should be placed on the major members involved within the activity. Unfortunately, a smartphone placed onto the body can also be non-compatible with some activities in an ecological environment (free-living conditions). Algorithm used for long recording periods can quickly consume the battery power, and may need a power supply. Another point consists of choosing the accurate available application.

### Mobile Applications

Smartphone applications experienced a boom in medical science. In 2016, the Play Store displayed 105,000 and the Apple Store 126,000 health or fitness-related apps (71). These applications propose physical exercises and fitness programs with or without connected objects such as wristband, pedometer, scale, HR monitor, smartphone, and smartwatch. When the mobile applications integrate the use of sensors (accelerometer, HR monitor, GPS), they inform the user of steps, distance, energy expenditure, speed, and heart frequency. The three most popular applications are Fitbit, Noom, and AppleHealth (Table 3). These special features are welcomed by the users. Conversely, most of the applications are not scientifically validated.

**Table 3:** Characteristics and physical activity parameters evaluated by the three most downloaded mobile applications.

**Application**	**Operating system**	**Wearable monitor**	**Measured parameters**
Fitbit	Android iOS Web	Accelerometer (wristband) Manual input	Number of steps or stairs Intensity Distance Calories burnt
Noom	Android iOS	Smartphone sensors GPS HR monitor	Distance Calories burnt Speed
Apple iHealth	iOS	RunKeeper (GPS) Moves (GPS and smartphone sensors) Manual input	Distance Calories burnt Number of steps Duration of activities

WellBeNet (eMouve) and IntellilifePro were two applications recently scientifically validated to assess accurately time spent in SB, LPA, MVPA, and the total energy expenditure associated. These two applications were specially developed to discriminate SB from LPA, such as standing or slow walking. Accelerometry data are collected via smartphones [WellBeNet (eMouve)] or via both a smartphone and smartwatch (IntellilifePro).

### E-Move

E-move (Android) application detects leg movements as the smartphone is worn in a front pants pocket. Different algorithms were designed for normal and overweight/obese adults. The total energy expenditure and time spent for each category of physical activity given by the E-Mouve algorithms were compared with reference method or device: either Armband or indirect calorimetry (FitmatePro, Cosmed). Absolute error of the total energy expenditure and activity estimates are 5.6 and 5.0%, respectively in normal weight volunteers, and 8.6 and 5.0% in overweight/obese participants (72, 73).

### IntellilifePro

IntellifePro is based on the simultaneous use of a smartphone and a smartwatch (Android or Apple) to detect both leg and wrist movements. IntellifePro can discriminate passive from active sitting when in a sitting posture, while the arm, the wrist and/or the hand are engaged in the movement. Absolute error of the total energy expenditure and activity estimates are 3.1, 2.8, 1.5, and 0.04%, for SB, light, moderate, and vigorous intensity, respectively. The absolute error for total energy expenditure was lower than 5% in free living conditions (74).

### Pressure Sensors

**Figure 4:**
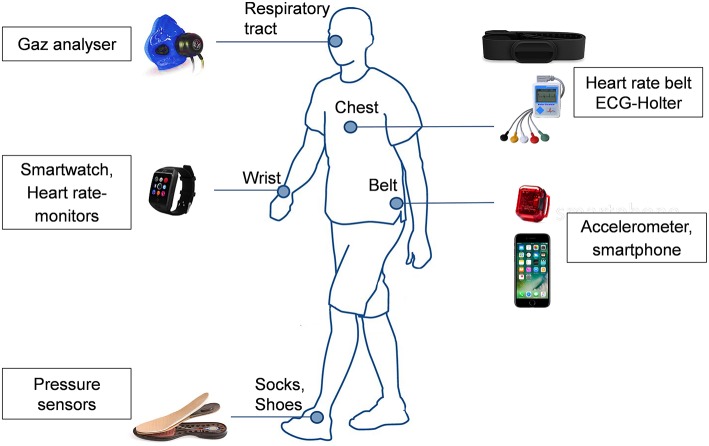
Current technologies to measure sedentary behavior and attachment on the body.

Another alternative to assess SB is via pressure sensors. Sensors can be placed in a sock, a shoe, or a chair. In a sock or shoe, a high pressure measured by the sensor is related to standing, and a low pressure is related to sitting or lying. On a chair, pressure sensors (sitting pad) are generally binary: active when the user is sitting, and inactive when nobody is sitting on the sensor (75). Current technologies and attachment on the body are presented in Figure 4.

### Characteristics of Sedentary Behavior

Total daily duration of SB is commonly used to study the effects on health of SB. However, characteristics of SB are of major importance on health. Particularly, continuous prolonged SB may be more deleterious on health outcomes than shorter bouts of SB but with the same duration (76, 77). The need for a definition of a sedentariness has also been proposed (78). Investigations of SB at work should not only assess total daily duration of SB, but also the patterns and durations of SB and non-SB periods. The context of SB is also important (what, where, why, when, and with whom).

### Limitations

Smart clothing (such as shirts with sensors measuring HR, socks or shoes combining pressure and accelerometers, or helmets and caps with a camera and GPS), goniometers (measuring an angle and angular position), electromyography meters (measuring the electrical activities of muscles EMG), and wearable camera have been voluntary excluded of the presented devices because still in development and not yet used to assess SB at work.

## Conclusion

We proposed a systematic review on tools available to measure SB at work. SB was mainly assessed by self-reported questionnaires or by only one wearable device. Studies using several devices were less common, and rarely studies used complex physiological systems. The wide range of wearable devices offer a variety of methods to evaluate SB at work. It is not an easy task to select the optimal device and the right measurement strategy for a particular study purpose. The main factors of work (inside or outside, working movements, and postures) and study population (i.e., number, age, gender, body mass index, and comorbidities) may also affect the choice. To assess SB at work, four determinants factors should be considered to choose the appropriate method: (1) quality of measure (e.g., time spent on SB or energy expenditure), (2) objectivity of the data and burden of workers (e.g., time/effort for measures), (3) cost/burden for the researcher, and (4) specific limitations due to environment and working activities. Available questionnaires are the most accessible method for a large population with a limited budget. SB at work (time sitting) is accessible from some specific items. It is also possible to deduct SB in measuring PA at work that is easily measurable. Assessments of SB need both measures of energy expenditure and of body posture (dual or multiple wearable devices with sensors). Accurate measure of SB at work need a sufficient number of subjects affected to the same assigned task and an objective measure coupled to a questionnaire (mixed approach method). For a restrictive group, SB at work can be objectively measured with wearable devices (accelerometers, heart-rate monitors, pressure meters, goniometers, electromyography meters, gas-meters) and can be associated with subjective measures (questionnaires). The number of devices worn increase the accuracy but make the analysis complex and time consuming. Furthers studies are necessary to improve the relative strengths and weakness of subjective or objective methods to assess SB at work.

## Author Contributions

FD conceived the article. GB, PC, DT, MD, and FD contributed to drafting the article. All authors performed critical revision of the article.

### Conflict of Interest Statement

FD established a public private partnership between the University Hospital of Clermont-Ferrand and WittyFit. However, he is not a member of the company and he is not paid by the company. The public private partnership only involves that he is the scientific leader, he owns all WittyFit data and can use it for research purposes. Therefore, as there is no money involved, the authors declare that the research was conducted in the absence of any commercial or financial relationships that could be construed as a potential conflict of interest.
