# Supplementary material for: How to Measure Sedentary Behavior at Work?
Source: Front Public Health. 2019 Jul 5;7:167. doi: 10.3389/fpubh.2019.00167 (PMC6633074; doi:10.3389/fpubh.2019.00167)
Supplement: Supplementary file 1 [file Table_1.pdf]

Table S1. Main characteristics of studies included in the systematic review, with methods to measure sedentariness (type of questionnaires and type of wearable devices, and main outcomes retrieved).

Study		Population (n total)	Type of study	Methods to measure sedentariness				Main measures of sedentariness
					Wearables devices			
First author, year	Title			Questionnaires	Category	Type	Other sensors	
Aittasalo et al 2017	Moving to business - Changes in physical activity and sedentary behavior after multilevel intervention in small and medium-size workplaces	396	Longitudinal	Other questionnaire	1. One common sensor	Accelerometer		Sitting and standing time/day, steps/day, sit/stand transitions
Albawardi et al 2017	Level of sedentary behavior and its associated factors among Saudi women working in office-based jobs in Saudi Arabia	420	Cross-sectional	Other questionnaire				Working sedentary and non-sedentary behaviors
Albawardi et al 2016	Levels and correlates of physical activity, inactivity and body mass index among Saudi women working in office jobs in Riyadh city	420	Cross-sectional	Other questionnaire				Sitting time
Alkatib et al 2013	Sedentary risk factors across genders and job roles within a university campus workplace: preliminary study	80	Cross-sectional	IPAQ				Working sitting time/week
Alkhajah et al 2012	Sit-stand workstations a pilot intervention to reduce office sitting time	33	Longitudinal		1. One common sensor	ActivPal3		Sitting time, breaks
Ariens et al 2001	Are neck flexion, neck rotation, and sitting at work risk factors for neck pain? Results of a prospective cohort study	686	Cross-sectional	Other questionnaire	1. One common sensor	Camera		Sitting time, steps/day
Bennie et al 2015	Total and domain-specific sitting time among employees in desk-based work settings in Australia	801	Cross-sectional	Other questionnaire				Sitting time, physical activity
Bort-Roig et al 2018	Monitoring sedentary patterns in office employees: validity of an m-health tool (Walk@Work-App) for occupational health	17	Cross-sectional		2. Mutliple sensors	Smartphone (Wow-App), ActivPal3TM, pedometer (YamaxSW200)		Woking and leisure sedentary behavior, physical activity
Boyle et al 2016	Sedentary work and the risk of breast cancer in premenopausal and postmenopausal women: a pooled analysis of two case-control studies	1762	Cross-sectional	Other questionnaire				Working, leisure sitting time
Boyle et al 2011	Long-term sedentary work and the risk of subsite-specific colorectal cancer	1939	Cross-sectional	Other questionnaire				Working, leisure, sitting, standing, time, sit-to-stand transitions
Brakenridge et al 2016	Organizational-level strategies with or without an activity tracker to reduce office workers' sitting time: rationale and study design of a pilot cluster-randomized trial	150	Longitudinal	Other questionnaire	1. One common sensor	LumoBack, ActivPal 3		Sitting time, steps counts
Brakenridge et al 2016	Evaluating the effectiveness of organisational-level strategies with or without an activity tracker to reduce office workers' sitting time: a cluster-randomised trial	153	Longitudinal		1. One common sensor	ActivPal3		Working, leisure, steps, sedentary behavior, physical activity

Brakenridge et al 2018	Evaluating short-term musculoskeletal pain changes in desk-based workers receiving a workplace sitting-reduction intervention	153	Longitudinal		1. One common sensor	ActivPal3	Working, leisure, sitting time, prolonged bouts of sitting (≥ 30 min), standing, and stepping time
Brisson et al 2000	Psychosocial factors at work, smoking, sedentary behavior, and body mass index: A prevalence study among 6995 white collar workers	6995	Cross-sectional	Other questionnaire			Working sitting time, standing time, steps counts
Brown et al 2003	Sitting time and work patterns as indicators of overweight and obesity in Australian adults	714	Cross-sectional	Other questionnaire	1. One common sensor	Pedometer	Working sitting, standing and walking time, sitting time over the whole day
Brown et al 2013	Objectively measured sedentary behavior and physical activity in office employees relationships with presenteeism	108	Cross-sectional		1. One common sensor	ActiGraph GT3X+	Working sitting, standing, stepping time, leisure physical activity
Bunan et al 2017	An intervention to reduce sitting and increase light-intensity physical activity at work: Design and rationale of the `Stand & Move at Work' group randomized trial	720	Longitudinal		1. One common sensor	Accelerometer	Working, leisure physical activity, sedentary
Calijouw et al 2017	RAAAF's office landscape The End of Sitting: Energy expenditure and temporary comfort when working in non-sitting postures	24	Longitudinal		3. Complex physiological systems	Indirect calorimetry, Cosmed K4b2	Working sitting time, steps, leisure total physical activity, light physical activity
Carr et al 2012	Feasibility of a portable pedal exercise machine for reducing sedentary time in the workplace	18	Longitudinal	Other questionnaire			Working sitting time, breaks, standing time, walking time, steps count
Carr et al 2016	Total worker health intervention increases activity of sedentary workers	54	Longitudinal	Other questionnaire			Working, leisure sitting, standing time, breaks, non-workday sitting, transport sitting, tv viewing
Chau et al 2014	The effectiveness of sit-stand workstations for changing office workers' sitting time: results from the Stand@Work randomized controlled trial pilot	42	Longitudinal	Other questionnaire	1. One common sensor	ActivPal	Working sitting time, longest period of sitting time, sit-to-stand transitions, light physical activity, stairs, walking
Chau et al 2011	A tool for measuring workers' sitting time by domain: the Workforce Sitting Questionnaire	95	Cross-sectional	WSQ			Sitting time, walking time
Chau et al 2012	cross-sectional associations between occupational and leisure-time sitting, physical activity and obesity in working adults	10785	Cross-sectional	Other questionnaire			Walking time, total and moderate physical activity

Chau et al 2012	Validity of the Occupational Sitting and Physical Activity Questionnaire	99	Cross-sectional	OSPAQ, MOSPA-Q	1. One common sensor	ActiGraph	Ecological momentary assessment (EMA)	Sitting, standing and walking time
Chia et al 2015	Office sitting made less sedentary - a future-forward approach to reducing physical inactivity at work	21	Longitudinal		1. One common sensor	ActiTrainer (accelerometer + HR recorder)		Working, leisure, sitting time
Cho et al 2013	Analysis according to gender and body mass index of the number of steps taken by sedentary workers as measured by a pedometer	36	Cross-sectional		1. One common sensor	Pedometer		Working sitting time, pedalling time, speed, distance, calories
Choi et al 2010	Sedentary work, low physical job demand, and obesity in us workers	2019	Cross-sectional	Other questionnaire				Sitting time
Choi et al 2016	24-hour work shifts, sedentary work, and obesity in male firefighters	308	Cross-sectional	Other questionnaire				Working sitting time
Clark et al 2011	Validity of self-reported measures of workplace sitting time and breaks in sitting time	121	Cross-sectional	Other questionnaire	1. One common sensor	Accelerometer		Working sitting time, light physical activity
Clemes et al 2014	Office workers' objectively measured sedentary behavior and physical activity during and outside working hours	170	Cross-sectional		1. One common sensor	ActiGraph GT1M		Sitting and standing time
Coenen et al 2017	Pre-existing low-back symptoms impact adversely on sitting time reduction in office workers	231	Longitudinal		1. One common sensor	ActivPal3TM		Working, leisure sitting time, number of breaks per day, physical activity
Cuthill et al 2008	Anaesthesia - a sedentary specialty? Accelerometer assessment of the activity level of anaesthetists while at work	45	Cross-sectional		1. One common sensor	Accelerometer		Sedentary breaks, physical activity
Danquah et al 2017	Take a Stand!-a multi-component intervention aimed at reducing sitting time among office workers-a cluster randomized trial	317	Longitudinal		1. One common sensor	ActiGraph GT3X (thigh)		Sitting time, physical activity
De Cocker et al 2015	Theory-driven, web-based, computer-tailored advice to reduce and interrupt sitting at work: development, feasibility and acceptability testing among employees	112	Longitudinal	Other questionnaire				Working sitting, standing, walking time
De Cocker et al 2016	The effectiveness of a web-based computer-tailored intervention on workplace sitting: a randomized controlled trial	213	Longitudinal	Other questionnaire				Energy expenditure, mets
De Cocker et al 2014	Understanding occupational sitting: Prevalence, correlates and moderating effects in Australian employees	993	Cross-sectional	WSQ				Working sitting, standing, walking, energy expenditure, kcal/h
Dollman et al 2016	Validity of self-reported sedentary time differs between Australian rural men engaged in office and farming occupations	57	Cross-sectional	Other questionnaire	1. One common sensor	Inclinometer		Working, leisure, steps, active time
Duncan et al 2015	Identifying correlates of breaks in occupational sitting: a cross-sectional study	5531	Cross-sectional	Other questionnaire				Working sitting, standing, bouts duration

Duncan et al 2013	Development and reliability testing of a self-report instrument to measure the office layout as a correlate of occupational sitting	37	Cross-sectional	Other questionnaire			Working, leisure, sitting, standing, physical activity time
Duning et al 2018	The effect of interrupting sedentary behavior on the cardiometabolic health of adults with sedentary occupations a pilot study	21	Longitudinal		2. Mutliple sensors	ActiGraph, ActivPal	Working sitting, standing, stepping, prolonged sitting bouts (≥ 30 min)
Dunstan et al 2013	Reducing office workers' sitting time: rationale and study design for the Stand Up Victoria cluster randomized trial	160	Longitudinal		1. One common sensor	ActivPal3	Sitting time, prolonged sitting, standing, stepping, moderate-to-vigorous physical activity, steps at work and daily
Edwardson et al 2018	A three arm cluster randomised controlled trial to test the effectiveness and cost- effectiveness of the SMART Work & Life intervention for reducing daily sitting time in office workers: study protocol	660	Longitudinal		1. One common sensor	ActivPal3 (wrist)	Working sitting , standing, stepping time
Evans et al 2012	Point-of-choice prompts to reduce sitting time at work: a randomized trial	28	Longitudinal		1. One common sensor	ActivPal	Working sitting time
Fisher et al 2018	Associations between the objectively measured office environment and workplace step count and sitting time: cross-sectional analyses from the active buildings study	131	Cross-sectional		1. One common sensor	ActivPal	Working sitting time, prolonged bouts (≥ 30 min), standing time, sit-to-stand transitions, walking time, leisure physical activity
Foley et al 2016	Sedentary behavior and musculoskeletal discomfort are reduced when office workers trial an activity-based work environment	88	Longitudinal	Other questionnaire	1. One common sensor	Accelerometer	Working, leisure, sitting time, prolonged bouts sitting time (≥ 30 min), time between sitting bouts, standing time, stepping time, number of steps
Gao et al 2016	Effects of environmental intervention on sedentary time, musculoskeletal comfort and work ability in office workers	45	Longitudinal	Other questionnaire			Working, leisure, sitting time, prolonged sitting
Gilson et al 2009	Do walking strategies to increase physical activity reduce reported sitting in workplaces: a randomized control trial	179	Longitudinal	Other questionnaire	1. One common sensor	Pedometer (YamaxSW200)	Sitting time (accumulated in bouts >= 30 min), standing, and moving
Gorman et al 2013	Does an `activity-permissive' workplace change office workers' sitting and activity time?	24	Longitudinal		1. One common sensor	ActivPal3	Sitting time and bouts > 30 min

Graves et al 2015	Evaluation of sit-stand workstations in an office setting: a randomised controlled trial	46	Longitudinal	Other questionnaire			Working sitting time
Gremaud et al 2018	Gamifying accelerometer use increases physical activity levels of sedentary office workers	144	Longitudinal		1. One common sensor	Fitbit Zip	Working sitting time, duration, breaks
Grunseit et al 2013	"Thinking on your feet": A qualitative evaluation of sit-stand desks in an Australian workplace	13	Longitudinal	Other questionnaire			Working sitting standing and stepping time
Gupta et al 2016	Prediction of objectively measured physical activity and sedentariness among blue-collar workers using survey questionnaires	214	Cross-sectional	Other questionnaire	1. One common sensor	Accelerometer	Working sitting time, number of sitting bouts > 20,30 and 55 min
Gupta et al 2016	What is the effect on obesity indicators from replacing prolonged sedentary time with brief sedentary bouts, standing and different types of physical activity during working days? a cross-sectional accelerometer-based study among blue-collar workers	692	Cross-sectional		1. One common sensor	ActivGraph GTX 3+ (thigh)	Working sitting time, number and duration of bouts > 30 min prolonged sitting
Gupta et al 2016	Are temporal patterns of sitting associated with obesity among blue-collar workers? A cross-sectional study using accelerometers	205	Cross-sectional		1. One common sensor	ActiGraphGT3X+ (thigh, trunk)	Sitting time, physical activity
Hadgraft et al 2017	Intervening to reduce workplace sitting: mediating role of social-cognitive constructs during a cluster randomised controlled trial	231	Longitudinal		1. One common sensor	ActivPal3	Sitting time, time of seat-cycle
Hall et al 2015	The effect of a sit-stand workstation intervention on daily sitting, standing and physical activity: protocol for a 12 month workplace randomised control trial	30	Longitudinal		2. Mutliple sensors	ActiGraph GTX3+,ActivPal 3	Working, leisure, sitting, standing, light physical activity time
Hallman et al 2015	Prolonged sitting is associated with attenuated heart rate variability during sleep in blue-collar workers	138	Longitudinal		2. Mutliple sensors	ActiGraph: GTX3, ActiHeart	Sitting time, night heart rate variability
Hallman et al 2016	Temporal patterns of sitting at work are associated with neck-shoulder pain in blue-collar workers: a cross-sectional analysis of accelerometer data in the DPHACTO study	659	Cross-sectional		1. One common sensor	ActiGraph GT3X (thigh, hip, trunk, upper dominant harm)	Working, leisure sitting time
Hallman et al 2015	Association between objectively measured sitting time and neck-shoulder pain among blue-collar workers	202	Cross-sectional		1. One common sensor	ActiGraph	Sitting and standing time physical activity /day
Hallman et al 2019	Objectively measured sitting and standing in workers: cross-sectional relationship with autonomic cardiac modulation	490	Cross-sectional		2. Mutliple sensors	Accelerometer, HR recorder	Working, leisure, sitting time, bouts > 30 min
Hallman et al 2016	Is prolonged sitting at work associated with the time course of neck-shoulder pain? A prospective study in Danish blue-collar workers	625	Cross-sectional		1. One common sensor	Accelerometer	Sedentary time, breaks, light and moderate to vigorous physical activity
Headley et al 2018	Subjective and objective assessment of sedentary behavior among college employees	127	Cross-sectional	OSPAQ	1. One common sensor	ActivPal3	Working, leisure, physical activity, moderate physical activity

Healy et al 2016	A cluster randomized controlled trial to reduce office workers' sitting time: effect on activity outcomes	231	Longitudinal		1. One common sensor	ActivPal3 TM	Moderate to vigorous physical activity (mets/day)
Healy et al 2013	Reducing sitting time in office workers: short-term efficacy of a multicomponent intervention	43	Longitudinal		1. One common sensor	ActivPal3	Sitting time, light, moderate to vigorous physical activity
Hendriksen et al 2016	Longitudinal relationship between sitting time on a working day and vitality, work performance, presenteeism, and sickness absence	502	Longitudinal	Other questionnaire			Steps/day, light physical activity, sedentary time
Honda et al 2014	Identifying associations between sedentary time and cardio-metabolic risk factors in working adults using objective and subjective measures: a cross-sectional analysis	661	Cross-sectional	Other questionnaire	1. One common sensor	Accelerometer	Occupational sitting-time
Hulsegge et al 2017	Shift workers have similar leisure-time physical activity levels as day workers but are more sedentary at work	200	Cross-sectional		1. One common sensor	ActiGraph GT3X (thigh)	Working, leisure, sitting time, walking, standing time
Hutchinson et al 2018	Changes in sitting time and sitting fragmentation after a workplace sedentary behaviour intervention	36	Longitudinal		1. One common sensor	ActivPal3	Working sitting time and whole day, physical activity job demand
Ishii et al 2018	Work engagement, productivity, and self-reported work-related sedentary behavior among japanese adults: a cross-sectional study	2572	Cross-sectional	Other questionnaire			Working sitting time, sitting travelling time, leisure sitting time, screen time
Jalayondeja et al 2017	Break in sedentary behavior reduces the risk of noncommunicable diseases and cardiometabolic risk factors among workers in a petroleum company	1133	Cross-sectional	Other questionnaire			Working, leisure sitting time, tv time, leisure physical activity
Jancey et al 2014	Application of the Occupational Sitting and Physical Activity Questionnaire (OSPAQ) to office based workers	99	Cross-sectional	OSPAQ	1. One common sensor	Accelerometer	Sitting breaks and duration
Jelsma et al 2019	The Dynamic Work study: study protocol of a cluster randomized controlled trial of an occupational health intervention aimed at reducing sitting time in office workers	250	Longitudinal	Other questionnaire	1. One common sensor	ActivPal	Working sedentary (sedentary occupations only; mixed occupations or non-sedentary occupations)
Johnsson et al 2017	Occupational sedentariness and breast cancer risk	29524	Cross-sectional	other-questionniare			Working sitting time
Josephson et al 2013	A sedentary job? Measuring the physical activity of emergency medicine residents	98	Cross-sectional		1. One common sensor	Pedometer	Working sedentary behaviour, working physical activity
Kayihan et al 2014	Relationship between daily physical activity level and low back pain in young, female desk-job workers	133	Cross-sectional	IPAQ-short			Working and leisure physical activity
Kazi et al 2014	A survey of sitting time among UK employees	504	Cross-sectional	Other questionnaire			Working, leisure sitting time

Keown et al 2018	Device-measured sedentary behavior patterns in office-based university employees	78	Cross-sectional		2. Mutliple sensors	ActiGraph GTX3+, ActivPal3	Working sitting time
Kikuchi et al 2015	Occupational sitting time and risk of all-cause mortality among Japanese workers	36516	Longitudinal	Other questionnaire			Working, transport, leisure sitting time, physical activity
Kirk et al 2016	Patterns of sedentary behaviour in female office workers	27	Cross-sectional	Other questionnaire	1. One common sensor	ActivPal T	Sedentary time, breaks number
Korshoj et al 2018	Is objectively measured sitting at work associated with low-back pain? A cross-sectional study in the DPhacto cohort	704	Cross-sectional		1. One common sensor	Accelerometer	Working, leisure sitting time, steps/hour, steps/day
Lamar et al 2016	Sedentary behavior in the workplace: a potential occupational hazard for radiologists	89	Cross-sectional	Other questionnaire	1. One common sensor	Fitbit One	Working sitting time, prolonged sitting time, screen time, driving time
Levine et al 2007	The energy expenditure of using a “walk-and-work” desk for office workers with obesity	15	Longitudinal		3. Complex physiological systems	Indirect calorimetry, Columbus Instruments	Average week working, week-end, sitting, standing, time, steps, sit to stand transitions
Li et al 2017	Reducing office workers' sitting time at work using sit-stand protocols: results from a pilot randomized controlled trial	26	Longitudinal	Other questionnaire	1. One common sensor	ActivPal	Working sitting time breaks from sitting (frequency/per work hour)
Lin et al 2018	A “Sit less, walk more” workplace intervention for office workers: long-term efficacy of a quasi-experimental study	101	Longitudinal	Other questionnaire			Working sedentary time, leisure and all day, moderate to vigorous physical activity
Lin et al 2017	Short-term efficacy of a “sit less, walk more” workplace intervention on improving cardiometabolic health and work productivity in office workers	99	Longitudinal	Other questionnaire			Working sedentary activities, sitting time
Lindsay et al 2016	Time kinetics of physical activity, sitting, and quality of life measures within a regional workplace: a cross-sectional analysis	346	Cross-sectional	IPAQ			Working sitting and standing time, TV time, leisure sitting time
Lunde et al 2017	Associations of objectively measured sitting and standing with low-back pain intensity: a 6-month follow-up of construction and healthcare workers	124	Cross-sectional		1. One common sensor	ActiGraph GT3X+ (thigh)	Working sedentary time, physical activity
Lynch et al 2013	A case-control study of lifetime occupational sitting and likelihood of breast cancer	2452	Cross-sectional	Other questionnaire			Working sitting time, breaks in sitting time, working sedentary time (hours per day, <100 counts per minute) and breaks per sedentary hour (number of times,

							>= 100 counts per minute)
Mackenzie et al 2015	Acceptability and feasibility of a low-cost, theory-based and co-produced intervention to reduce workplace sitting time in desk-based university employees	17	Longitudinal	Other questionnaire			Working sitting time, occupational energy expenditure, leisure physical activity
Mainsbridge et al 2014	The effect of an e-health intervention designed to reduce prolonged occupational sitting on mean arterial pressure	29	Longitudinal	OSPAQ			Working, leisure and transport-relayed sitting time, screen time
Mansoubi et al 2016	Using sit-to-stand workstations in offices: is there a compensation effect?	40	Longitudinal		2. Mutliple sensors	ActiGraphGT3X+, ActivPal	Working sitting, standing, walking, or heavy labour time, transport-related walking, leisure-time sitting and physical activity
Matic et al 2011	Smart phone sensing to examine effects of social interactions and non-sedentary work time on mood changes	9	Cross-sectional		1. One common sensor	Smartphone	Working standing and sitting posture
Matsuo et al 2016	Percentage-method improves properties of Workers' Sitting-and Walking-Time Questionnaire	65	Cross-sectional	WSQ	1. One common sensor	ActivPal	Worrking sitting time
McCrady et al 2009	Sedentariness at work: how much do we really sit?	21	Cross-sectional		2. Mutliple sensors	Physical Activity Monitoring System: 4 inclinometers, 2 accelerometers (attached to the torso, thigh, and trunk using unique undergarments)	Working sitting, tv time
Miyachi et al 2015	Installation of a stationary high desk in the workplace: effect of a 6-week intervention on physical activity	32	Longitudinal		1. One common sensor	Accelerometer	Working sedentary behavior, job strain
Moerl et al 2013	Lumbar posture and muscular activity while sitting during office work	13	Cross-sectional		1. One common sensor	sEMG	Sitting time, breaks
Moller et al 2016	Multi-wave cohort study of sedentary work and risk of ischennic heart disease	145850	Longitudinal	Other questionnaire			Working and leisure sitting, standing, walking, carrying loads time
Moreno-Franco et al 2015	Association between daily sitting time and prevalent metabolic syndrome in an adult working population: the AWHS cohort	1415	Cross-sectional	Other questionnaire			Work-related sedentary behavior

Mullane et al 2017	Social ecological correlates of workplace sedentary behavior	478	Cross-sectional		1. One common sensor	ActivPal	Working and leisure sitting time, physical activity
Munir et al 2015	Work engagement and its association with occupational sitting time: results from the Stormont study	4436	Cross-sectional	Other questionnaire			Working sitting time
Nagaya et al 2001	Effects of sedentary work on physical fitness and serum cholesterol profile in middle-aged male workers	1117	Cross-sectional	Other questionnaire			Sedentary work and low physical activity job demand
Neuhaus et al 2014	Workplace sitting and height-adjustable workstations a randomized controlled trial	34	Longitudinal		1. One common sensor	ActivPal3	Sedentary work, physical activity
Neuhaus et al 2014	Iterative development of Stand Up Australia: a multi-component intervention to reduce workplace sitting	32	Longitudinal		1. One common sensor	ActivPal3	Working time spend in sedentary position
Nooijen et al 2019	Improving office workers' mental health and cognition: a 3-arm cluster randomized controlled trial targeting physical activity and sedentary behavior in multi-component interventions	330	Longitudinal	Other questionnaire	2. Mutliple sensors	Accelerometer, inclinometer	Working, leisure and transport sitting time and physical activity
O'Connell et al 2015	Providing NHS staff with height-adjustable workstations and behaviour change strategies to reduce workplace sitting time: protocol for the Stand More AT (SMArT) Work cluster randomised controlled trial	238	Longitudinal		1. One common sensor	ActivPal	Sitting time
Oliver et al 2010	Utility of accelerometer thresholds for classifying sitting in office workers	21	Cross-sectional		2. Mutliple sensors	ActiCal, ActvPal	Working sitting time, leisure, time commuting
Olsen et al 2018	A brief self-directed intervention to reduce office employees' sedentary behavior in a flexible workplace	30	Longitudinal	Other questionnaire			Working, leisure and transport-relayed physical activity, sitting time,
Olsen et al 2018	Flexible work: the impact of a new policy on employees' sedentary behavior and physical activity	24	Cross-sectional	Other questionnaire			Working and day sitting time
Parry et al 2013	Participatory workplace interventions can reduce sedentary time for office workers-a randomised controlled trial	62	Longitudinal		1. One common sensor	ActiGraph GT3X	Physical activity, physical inactivity
Pedersen et al 2016	Is self-reporting workplace activity worthwhile? Validity and reliability of occupational sitting and physical activity questionnaire in desk-based workers	236	Longitudinal	IPAQ	1. One common sensor	ActivPal	Walking steps/day
Pedersen et al 2016	Intra-individual variability in day-to-day and month-to-month measurements of physical activity and sedentary behaviour at work and in leisure-time among Danish adults	135	Cross-sectional		1. One common sensor	ActiGraphGT3X+ (thigh)	Sedentary time, prolonged sedentary time
Pedisic et al 2014	Workplace Sitting Breaks Questionnaire (SITBRQ): an assessment of concurrent validity and test-retest reliability	147	Cross-sectional	SITBRQ (Workplace Sitting Breaks Questionnaire)	1. One common sensor	ActiGraph GT1M	Working physical activity, leisure physical activity, estimated energy expenditure

Pereira et al 2012	Sedentary behaviour and biomarkers for cardiovascular disease and diabetes in mid-life: the role of television-viewing and sitting at work	7660	Cross-sectional	Other questionnaire			Working physical activity, sedentary leisure time activity
Picavet et al 2016	The Relation between occupational sitting and mental, cardiometabolic, and musculoskeletal health over a period of 15 years - The Doetinchem cohort study	1509	Longitudinal	Other questionnaire			Working sitting time
Pontt et al 2015	Comparison of sedentary behaviours among rural men working in offices and on farms	116	Cross-sectional		1. One common sensor	ActivPal (thigh)	Working sitting, standing, walking time
Priebe et al 2015	Less sitting and more moving in the office: using descriptive norm messages to decrease sedentary behavior and increase light physical activity at work	142	Longitudinal	Other questionnaire			Working, leisure, time spent sitting, standing, stepping, and total number of steps
Puig-Ribeira et al 2017	Impact of a workplace `sit less, move more' program on efficiency-related outcomes of office employees	264	Longitudinal	Other questionnaire	1. One common sensor	Pedometer	Working sitting, standing and walking time
Puig-Ribera et al 2015	Patterns of impact resulting from a `Sit less, move more' web-based program in sedentary office employees	263	Longitudinal	IPAQ-short			Occupational, leisure, total energy expenditure, energy expenditure by occupational category (light, moderate, heavy)
Puig-Ribera et al 2015	Self-reported sitting time and physical activity: interactive associations with mental well-being and productivity in office employees	557	Cross-sectional	IPAQ-short			Working sitting time, physical activity and while travelling on weekend
Radas et al 2013	Evaluation of ergonomic and education interventions to reduce occupational sitting in office-based university workers: study protocol for a randomized controlled trial	60	Longitudinal	OSPAQ	1. One common sensor	ActiGraph GT3X	Low, moderate and high physical activity/day, met score
Rodriguez-Hernandez et al 2019	The effect of 2 walking programs on aerobic fitness, body composition, and physical activity in sedentary office employees	24	Longitudinal		1. One common sensor	Accelerometer (wrist)	Working sitting, standing, walking and vigorous activities
Ryan et al 2011	Sitting patterns at work: objective measurement of adherence to current recommendations	83	Longitudinal		1. One common sensor	ActivPal TM	Sitting time, walking time, vigorous and total physical activity
Ryde et al 2012	Validation of a novel, objective measure of occupational sitting	13	Cross-sectional		2. Mutliple sensors	Sitting pad, ActivPal3, ActiGraph GT3X, camera	Working sitting, standing, walking and vigorous activities
Ryde et al 2013	Desk-based occupational sitting patterns weight-related health outcomes	105	Cross-sectional		2. Mutliple sensors	Sitting pad, ActiGraph GT3X	Working siiting time, steps counts
Saidj et al 2015	Descriptive study of sedentary behaviours in 35,444 French working adults: cross-sectional findings from the ACTI-Cites study	35444	Cross-sectional	Other questionnaire			Working and leisure sedentary behavior

Saidj et al 2013	Separate and joint associations of occupational and leisure-time sitting with cardio-metabolic risk factors in working adults: a cross-sectional study	2544	Cross-sectional	Other questionnaire			Working sitting time, transition
Schuna et al 2014	Evaluation of a workplace treadmill desk intervention: a randomized controlled trial	41	Longitudinal		1. One common sensor	Accelerometer	Working sitting time and breaks, sit-to-stand transitions
Schwartz et al 2016	Effect of a novel two-desk sit-to-stand workplace (ACTIVE OFFICE) on sitting time, performance and physiological parameters: protocol for a randomized control trial	18	Longitudinal	IPAQ-long			Working upper limb movement, emg, heart rate, estimated energy expenditure
Sharma et al 2018	A quantitative evaluation of electric sit-stand desk usage: 3-month in-situ workplace study	364	Cross-sectional				Working sitting, standing and walking time
Simons et al 2013	Physical activity, occupational sitting time, and colorectal cancer risk in the netherlands cohort study	120852	Cross-sectional	Other questionnaire			Working and leisure sitting, walking time, walking velocity, daily energy estimation
Sisson et al 2009	Leisure time sedentary behavior, occupational/domestic physical activity, and metabolic syndrome in us men and women	3556	Cross-sectional	Other questionnaire			Working sitting time, frequency and duration leisure physical activity
Sitthipornvorakul et al 2015	The effect of daily walking steps on preventing neck and low back pain in sedentary workers: a 1-year prospective cohort study	387	Cross-sectional	Other questionnaire			Steps by 12h-shift
Smith et al 2015	Weekday and weekend patterns of objectively measured sitting, standing, and stepping in a sample of office-based workers: the active buildings study	164	Longitudinal				
Stamatakis et al 2012	Sedentary time in relation to cardio-metabolic risk factors: differential associations for self-report vs accelerometry in working age adults	1150	Cross-sectional	Other questionnaire	1. One common sensor	Accelerometer	Sedentary time (h/day), sedentary bouts (h/day), length of work sedentary bouts (min), number of breaks in sedentary behaviour at work (per day), length of breaks in sedentary behaviour at work (min),number of steps per hour at work
Stoy et al 2004	Semen quality and sedentary work position	1747	Cross-sectional	Other questionnaire			Working sitting, standing, light physical activity, stepping cadence < 100 steps/min), and prolonged sitting bouts (> 30 min)

Sturgeon et al 2017	Nurse Educators' Occupational and Leisure Sitting Time	56	Cross-sectional	WSQ			Working steps and % sitting time/h
Sudholz et al 2018	Reliability and validity of self-reported sitting and breaks from sitting in the workplace	39	Cross-sectional	Other questionnaire	2. Mutliple sensors	ActiGraph , ActivPal	Working, leisure, sedentary time (sit and lie), brief (≤ 5 mins), moderate (>5 and ≤ 30 mins), and long (>30mins) bouts, standing time, walking tiem, moderate to vigorous physical activity time
Taylor et al 2016	Impact of booster breaks and computer prompts on physical activity and sedentary behavior among desk-based workers: a cluster-randomized controlled trial	175	Longitudinal	Other questionnaire	1. One common sensor	Pedometer	Working, leisure, sitting time, prolonged sitting (≤ 5 mins), (>5 and ≤ 30 mins), (>30mins) bouts, moderate to vigorous physical activity
Tissot et al 2005	Standing, sitting and associated working conditions in the Quebec population in 1998	9425	Cross-sectional	Other questionnaire			Working, leisure, sitting , standing time, steps, moderate to vigorous physical activity (MVPA)
Tsurumi et al 2002	Estimation of energy expenditure during sedentary work with upper limb movement	12	Cross-sectional		2. Mutliple sensors	sEMG, accelerometer, HR recorder	Working, leisure, light-intensity, moderate-to-vigorous physical activity, time lying, standing, stepping, prolonged sedentary bouts, breaks in sedentary time
Tudor-Locke et al 2009	Leisure-time physical activity and occupational sitting: Associations with steps/day and BMI in 54-59 year old Australian women	158	Cross-sectional		1. One common sensor	Pedometer	Working, leisure sitting, standing time
Uijdewilligen et al 2017	Correlates of occupational, leisure and total sitting time in working adults: results from the Singapore multi-ethnic cohort	9384	Cross-sectional	Other questionnaire			Working sedentary time (<= 150 counts) and light physical activity (151 to 1689 counts)
Van Dommelen et al 2016	Objectively measured total and occupational sedentary time in three work settings	205	Cross-sectional		1. One common sensor	ActiGraph	Working, leisure, sitting time, (brief (0-5 min), moderate (> 5-20 min) and prolonged (> 20

							min) bouts), physical activity, working, leisure sitting with upper arm over 90° (h/day)
Van Dyck et al 2015	The contribution of former work-related activity levels to predict physical activity and sedentary time during early retirement: moderating role of educational level and physical functioning	392	Cross-sectional	IPAQ			Working, leisure, sitting time, prolonged sitting bouts ≥30 min, total physical activity, light and moderate physical activity
Van Nassau et al 2015	Validity and responsiveness of four measures of occupational sitting and standing	42	Longitudinal	OSPAQ, WSQ	2. Mutliple sensors	ActiGraph, ActivPal	Working and leisure time spent sedentary (<100 cpm), in light-intensity physical activity (100 to 1951 cpm), and moderate-to-vigorous physical activity (1952 cpm), and non working and non workdays
Vandelanotte et al 2013	Associations between occupational indicators and total, work-based and leisure-time sitting: a cross-sectional study	1194	Cross-sectional	WSQ			Working, leisure, sedentary time, light, moderate to vigorous physical activity, prolonged sitting bouts (≥ 30 min)
Vaz et al 2004	How sedentary are people in `sedentary' occupations? The physical activity of teachers in urban South India	198	Cross-sectional	Other questionnaire			Siting time, at work and leisure
Verweij et al 2012	The application of an occupational health guideline reduces sedentary behaviour and increases fruit intake at work: results from a randomized controlled trial	523	Longitudinal	Other questionnaire			Working sitting time
Wallmann-Sperlich et al 2014	Socio-demographic, behavioural and cognitive correlates of work-related sitting time in German men and women	1515	Cross-sectional	Other questionnaire			Working sitting, standing time, leisure sitting time, physical activity, heart rate, hrv
Waters et al 2016	Assessing and understanding sedentary behaviour in office-based working adults: a mixed-method approach	33	Cross-sectional	Other questionnaire	1. One common sensor	Accelerometer	Working, leisure, sitting time, sitting bouts ≥ 5 min, >5-≤20min, >20min
Wong et al 2014	Patterns and perceptions of physical activity and sedentary time in male transport drivers working in regional Australia	23	Cross-sectional		1. One common sensor	Accelerometer	Working sitting time

Yao et al 2002	Energy requirements of urban Chinese adults with manual or sedentary occupations, determined using the doubly labeled water method	73	Cross-sectional	MOSPA-Q	3. Complex physiological systems	Double labeled water		Working sitting, standing, walking time, steps
Yip et al 2004	New low back pain in nurses: work activities, work stress and sedentary lifestyle	144	Cross-sectional	Other questionnaire				Working sedentary (<150 cpm),pa: light (151-2,689 cpm) and moderate+ (2,690 cpm) and off-work
Zhu et al 2018	Long-term effects of sit-stand workstations on workplace sitting: a natural experiment	36	Longitudinal		1. One common sensor	ActivPal3c	Electric-sit-stand desk computer monitoring	Working sitting or standing position, frequency of transition
