# Supplementary material for: How to Measure Sedentary Behavior at Work?
Source: Front Public Health. 2019 Jul 5;7:167. doi: 10.3389/fpubh.2019.00167 (PMC6633074; doi:10.3389/fpubh.2019.00167)
Supplement: Supplementary file 2 [file Data_Sheet_1.PDF]

Appendix 1
Details for the search strategy used within each database

Pubmed

(measure*[TW] OR measuring[TW] OR questionnaire*[TW])

AND
("work"[MH] OR "labor"[TW] OR "job"[TW] OR work[tw] OR works[tw] OR work's[tw] OR Worker*[TW] OR working[TW] OR Worksite*[TW] OR workstation*[TW] OR "workplace"[MH] OR workplace*[TW] OR occupation*[tw] OR employe*[tw])

AND
("Sedentary Behavior"[MH] OR "sedentary"[TW] OR "sedentariness"[TW] OR seated posture[TW] OR "Seated Position"[TW] OR desk[tiab] OR (office[TW] AND (inactiv*[TW] OR seated[TW])) OR "Sitting Position"[MH] OR "sitting"[TW])

NOT ((animals [mh] NOT humans [mh]))

NOT (pregnancy[Mesh] OR pregnancy[Title])

NOT occupational musculoskeletal disorders[tiab]

NOT ("child"[Title] OR "childs"[Title] OR "children"[Title] OR "Offspring"[Title] OR "minor"[Title] OR "adolescent"[Title] OR "adolescents"[Title] OR "teens"[Title] OR "teenagers"[Title] OR "teenager"[Title] OR "adolescence"[Title] OR "teen"[Title])

Filter Language = English
Filter Dates = 2000-2019

CENTRAL

measure*:*ti,ab,kw* OR measuring:*ti,ab,kw* OR questionnaire*:*ti,ab,kw*

AND
work:*ti,ab,kw* OR labor:*ti,ab,kw* OR job:*ti,ab,kw* OR works:*ti,ab,kw* OR work's:*ti,ab,kw* OR Worker*:*ti,ab,kw* OR working:*ti,ab,kw* OR Worksite*:*ti,ab,kw* OR workstation*:*ti,ab,kw* OR workplace*:*ti,ab,kw* OR occupation*:*ti,ab,kw* OR employe*:*ti,ab,kw*

AND
"sedentary behavior":ti,ab,kw OR sedentary:ti,ab,kw OR sedentariness:ti,ab,kw OR "seated posture":ti,ab,kw OR "seated position":ti,ab,kw OR desk:ti,ab,kw OR (office:ti,ab,kw AND (inactiv*:ti,ab,kw OR seated:ti,ab,kw)) OR sitting:ti,ab,kw
NOT

child:*ti* OR childs:*ti* OR children:*ti* OR Offspring:*ti* OR minor:*ti* OR adolescent:*ti* OR adolescents:*ti* OR teens:*ti* OR teenagers:*ti* OR teenager:*ti* OR adolescence:*ti* OR teen:*ti*

NOT "occupational musculoskeletal disorders":*ti,ab,kw*

NOT pregnancy:*ti*

Filter Language = not available in CENTRAL
Filter Dates = 2000-2019

Web of science (WOS)

TS=(measure* OR measuring OR questionnaire*) OR TI =(measure* OR measuring OR questionnaire*)

AND
TI=(work OR labor OR job OR work OR works OR work's OR Worker* OR working OR Worksite* OR workstation* OR workplace OR workplace* OR occupation* OR employe*)

AND
TI=("Sedentary Behavior" OR sedentary OR sedentariness OR "seated posture" OR "Seated Position" OR "Sitting Position" OR sitting OR desk) OR TI=(office AND (inactiv* OR seated))

NOT
TI=("occupational musculoskeletal disorders" OR pregnancy OR child OR childs OR children OR Offspring OR minor OR adolescent OR adolescents OR teens OR teenagers OR teenager OR adolescence OR teen)

Filter Language = English
Filter Dates = 2000-2019
Databased restricted to:
- Science Citation Index Expanded (SCI-EXPANDED)
- Conference Proceedings Citation Index- Science (CPCI-S)
- Book Citation Index– Science (BKCI-S)
- Emerging Sources Citation Index (ESCI)

Embase

('worker'/exp OR 'work'/exp OR occupational)

AND ('sitting'/exp OR 'sedentary lifestyle'/exp)

AND ('measurement'/exp OR 'questionnaire'/exp)
